# Microbial Metabolism of Naringin and the Impact on Antioxidant Capacity

**DOI:** 10.3390/nu14183765

**Published:** 2022-09-13

**Authors:** Xuan Zeng, Yuying Zheng, Yan He, Jiashuo Zhang, Wei Peng, Weiwei Su

**Affiliations:** Guangdong Engineering & Technology Research Center for Quality and Efficacy Reevaluation of Post-Market Traditional Chinese Medicine, Guangdong Provincial Key Laboratory of Plant Resources, School of Life Sciences, Sun Yat-sen University, Guangzhou 510275, China

**Keywords:** naringin, rat microbiota, metabolism, antioxidant capacity

## Abstract

Naringin is a dietary flavonoid glycoside with broad bioactivities, and it has been found to undergo extensive microbial metabolism in human gut. Microbial metabolites are believed to play an important role in the overall bioactivity of naringin. However, knowledge is scarce about its microbial metabolism in laboratory rats, which are the most commonly used animal model for naringin-related biomedical studies. Herein, we profiled the microbial metabolism of naringin in rat by an in vitro anaerobic fermentation combined with LC-MS/MS methods. A total of 35 microbial metabolites were identified, and corresponding metabolic pathways were proposed. Naringin and its metabolites were further quantified in fermentation samples. Rhoifolin, neoeriocitrin, neohesperidin, naringenin, methylated naringin, and hydroxylated naringin were detected as the primary microbial metabolites. Moreover, antioxidant capacity assays suggested that fermentation-associated microbial metabolites exhibited higher antioxidant activity than original naringin. Obtained results contribute to a more comprehensive understanding of the microbial metabolism and antioxidant capacity of naringin.

## 1. Introduction

Naringin, chemically known as 5,7,4′-trihydroxyflavanone-7-*O*-neohesperidoside, is a dietary flavonoid glycoside abundant in citrus juice [1]. It contributes the distinct bitter taste to citrus juice, in which the content could reach 84.8 mg/L [2,3]. Naringin has been explored broadly for its biological and pharmacological effects. It has been documented to relieve oxidative stress [4], inflammatory response [5], neurological disorder [6], cardiovascular dysfunction [7], metabolic syndrome [8], and respiratory disease [9]. In recent years, naringin has been increasingly used as a phytopharmaceutical in dietary supplement formulations [10].

Gut-microbiota-related gastrointestinal digestion is believed to contribute significantly to the bioavailability of dietary polyphenols [11], as well as their physiological function (antioxidant [12], antimicrobial [13], and anti-inflammatory activities [14]). To date, a number of studies have reported the metabolic profiles of naringin in vitro and in vivo [9,15]. In the gastrointestinal tract, microbial enzymes could eliminate the glycoside of naringin to yield its aglycone naringenin, metabolize it into other flavonoid metabolites, and further degrade it into a variety of ring-fission products [16,17,18]. Recent studies have revealed the important role of these microbial metabolites in the overall bioactivity of naringin [19,20,21]. In fact, the physiological effects of naringin cannot be fully achieved in the absence of microbial metabolites due to the extensive biotransformation of parent compound [22]. Therefore, it is important to profile the gut microbial metabolism of naringin.

In vitro anaerobic fermentation is a practical method to investigate gut microbiota-mediated metabolism of natural products, such as flavonoids [23], ginsenosides [24], and theaflavins [25]. To date, several studies have reported the human intestinal microbial metabolism of naringin [26,27,28]. However, information concerning the microbial metabolism of naringin in laboratory animals is very limited. Due to limitations of human research, the use of laboratory animals remains essential in biomedical studies. The laboratory rat is the first mammalian species to be domesticated for scientific purposes, and it has become a commonly used animal model in biomedical research [29]. According to PubMed search results, there are nearly 500 reports focused on the biological and pharmacological actions of naringin in rats, far more than other laboratory animals. A comprehensive characterization of the microbial metabolism of naringin is a critical prerequisite to understand the physiological effects and mechanism in rat, owing to the important contribution of microbial metabolites to overall bioactivity. Nevertheless, our knowledge about the metabolism of naringin by rat gut microbiota is scarce. Here, we investigated the microbial metabolism of naringin in rat by an in vitro anaerobic fermentation combined with LC-MS/MS methods. The impact of microbial metabolism on the antioxidant capacity was also evaluated. Obtained results would provide further knowledge about the microbial metabolism of naringin, and give an insight into the important role of microbial metabolites in the overall bioactivity of naringin in clinical practice.

## 2. Materials and Methods

### 2.1. Chemicals and Reagents

The standard substances of naringin (purity: 95.0%), naringenin (purity: 95.0%), hesperetin (purity: 95.0%), and *p*-coumaric acid (purity: 98.0%) were acquired from Sigma-Aldrich (St. Louis, MI, USA). Rhoifolin (purity: 98.0%) and eriodictyol (purity: 98.0%) were acquired from Meilunbio (Dalian, China). Neoeriocitrin (purity: 99.0%) was obtained from Cayman (Ann Arbor, MI, USA). Neohesperidin (purity: 99.0%) and apigenin (purity: 99.0%) were purchased from Macklin (Shanghai, China). Caffeic acid (purity: 99.7%) and isoquercitrin (purity: 97.2%) were purchased from National Institute for the Control of Pharmaceutical and Biological Products (Beijing, China). BL agar medium base was obtained from G-CLONE (Beijing, China) and is composed of dipotassium hydrogen phosphate, sodium chloride, potassium dihydrogen phosphate, ammonium sulfate, calcium chloride, magnesium sulfate, resazurin, L-cysteine, L-ascorbic acid, sodium carbonate, beef meal, peptone, and agar.

LC-MS-grade methanol was acquired from Fisher Scientific (Fair Lawn, NJ, USA). HPLC-grade acetonitrile was purchased from Honeywell B&J Chemicals (Morristown, NJ, USA). LC-MS-grade formic acid was acquired from Sigma-Aldrich (St. Louis, MI, USA). Water was purified with a Milli-Q system (Millipore Corporation; Billerica, MA, USA) and further filtered through a microfiltration membrane (0.22 μm) before use.

### 2.2. Animals

Six healthy male Sprague Dawley rats (weighing 180–220 g) were acquired from Guangdong Medical Laboratory Animal Center (Guangzhou, China). Animals were housed in a controlled environment (20–25 °C, 55 ± 15% relative humidity, and 12 h light/dark cycles), and fed a low-flavonoid diet with water available ad libitum. All experimental operations were carried out according to the Guide for the Care and Use of Laboratory Animals (8th edition) issued by National Academies Press, and approved by the Institutional Animal Care and Use Committee (IACUC) in Sun Yat-Sen University.

### 2.3. In Vitro Fermentation of Naringin with Rat Gut Microbiota

The in vitro fecal fermentation of naringin was conducted in the light of the methodology reported by Ma et al. [30], with some optimizations. Fecal materials were collected from the small intestinal and caecal lumen, processed separately, and used for subsequent fermentation assays to investigate whether there were differences in the metabolic capacity of intestinal microbes at different locations. After collection, fecal material (1.0 g) was accurately weighed and homogenized with cold saline in a ratio of 1:4 (*w*/*v*) to afford fecal slurry. The fecal slurry was centrifuged at 5000× *g* for 30 min at 4 °C to obtain corresponding rat fecal suspension. Subsequently, an aliquot of 190 μL rat fecal suspension was spiked with 1710 μL fluid medium (obtained by dissolving the agar medium base in water and then autoclaving at 121 °C) and 100 μL naringin solution (prepared by dissolving the solid naringin in methanol at a concentration of 10 μmol/mL). The final concentrations of naringin were 500 nmol/mL. The mixture was then incubated at 37 °C for 12 h in an anaerobic condition. To identify and quantify the metabolites derived from naringin, aliquots of 100 μL samples were taken after 12 h fermentation and immediately vortex-mixed with 200 μL cold acetonitrile (containing internal standard at a concentration of 1 μg/mL) for 3 min. After centrifugation (15,000× *g*, 30 min, 4 °C), the supernatants were collected and stored at −70 °C until LC-MS/MS analysis.

### 2.4. Identification of the Microbial Metabolites of Naringin by UHPLC-Q-TOF-MS/MS

To identify the microbial metabolites of naringin, obtained supernatants were analyzed with a UHPLC system (Nexera X2; Shimadzu, Tokyo, Japan) tandem hybrid triple quadrupole time-of-flight mass spectrometer (Triple TOF 5600 plus; Sciex, Foster City, FL, USA) equipped with an electrospray ionization (ESI) source. Detailed parameters of chromatographic separation and MS/MS detection were the same as our preliminary studies [18,31]. Instrument operation and data acquisition were performed with Analyst software (version TF 1.6; Sciex, Foster City, FL, USA), while the data processing was conducted with PeakView software (version 1.2; Sciex, Foster City, FL, USA).

### 2.5. Quantification of the Microbial Metabolites of Naringin by UHPLC-Q-Trap-MS/MS

To quantitatively profile the microbial metabolism of naringin in rat fecal fermentation samples, a UHPLC-Q-Trap-MS/MS was developed and validated for the simultaneous determination of naringin and its primary metabolites, including rhoifolin, neoeriocitrin, neohesperidin, naringenin, apigenin, eriodictyol, hesperetin, *p*-coumaric acid, and caffeic acid. Chromatographic separation was conducted with a UHPLC system (XR; Shimadzu, Tokyo, Japan) equipped with a communications bus module, a degassing unit, a binary pump, an autosampler, and a column oven. The separation of metabolites was carried out on an ACQUITY UPLC^®^ BEH C_18_ column (50 mm × 2.1 mm, 1.7 μm; Waters, Milford, DE, USA) with the column temperature operated at 40 °C. Mobile phases consisting of water (A) and methanol (B), both containing 0.1% formic acid (*v*/*v*), were employed at a flow rate of 0.4 mL/min. Mobile phases were eluted as the following linear gradient elution program: 5% to 35% B at 0–7 min, 35–100% B at 7–11 min, and a 4 min post-run time for equilibration. The temperature of autosampler was maintained at 15 °C, and the injection volume was set as 10 μL.

Following the chromatographic separation, UHPLC effluent was directly introduced to a hybrid triple quadrupole linear ion trap mass spectrometer (Q-Trap 6500^+^; Sciex, Foster City, CA, USA) equipped with an ESI source. The mass spectrometer was operated in negative ion mode and multiple reactions monitoring (MRM) scan mode. Nitrogen was engaged as auxiliary gas, curtain gas, and collision gas. The common parameters of the mass spectrometer were set as follows: ion spray voltage, −4500 V; atomization temperature, 550 °C; ion source gas 1, 55 psi; ion source gas 2, 55 psi; curtain gas, 35 psi; collision gas, medium level. The MRM transitions, declustering potentials (DP), and collision energies (CE) were optimized as Table 1. Raw MS/MS data were acquired with Analyst software (version TF 1.7 with HotFix 3; Sciex, Foster City, CA, USA) and then processed with OS software (version 1.4; Sciex, Foster City, CA, USA).

The developed method was further validated for specificity, carry-over effect, calibration curve, lower limit of quantification (LLOQ), precision, accuracy, matrix effect, dilution integrity (20 times), and stability (long-term cryopreserved at −70 °C for 1 month, incubation at 37 °C for 12 h, placement in autosampler at 15 °C for 24 h) in light of the Guidelines for Validation of Quantitative Analytical Method of Biological Samples documented in the Pharmacopoeia of the People’s Republic of China (ChP) of 2020 edition [32]. The detailed experimental processes of methodology validation referred to our reported studies [33,34].

### 2.6. Measurement of Total Antioxidant Capacity of Naringin and Its Primary Metabolites

In this work, the quantitative results in the cecal contents group were chosen to evaluate the impact of microbial metabolism on the antioxidant capacity of naringin. The pre-fermented working solution of naringin was prepared at the concentration of 363.4 nmol/mL. The post-fermented working solution was prepared by spiking the stock solution of naringin and its metabolites rhoifolin, neoeriocitrin, neohesperidin, naringenin, apigenin, eriodictyol, hesperetin, *p*-coumaric acid, and caffeic acid in the determined proportion, so as to obtain a total molarity the same as the pre-fermented working solution of naringin. The total antioxidant capacity (TAOC) of the above two working solutions were evaluated with ABTS (2,2′-azino-bis (3-ethylbenzothiazoline-6-sulfonate) radical scavenging assay, ferric reducing antioxidant power (FRAP) assay, DPPH (α,α-diphenyl-β-picrylhydrazyl) radical scavenging assay, and hydroxyl radical (∙OH) scavenging assay, following the protocols of corresponding kits purchased from Nanjing Jiancheng Bioengineering Institute (Nanjing, China).

### 2.7. Data Analysis

Raw LC-MS/MS data were acquired with SCIEX Analyst software (Version 1.7) and then processed with SCIEX OS software (Version 1.4). Data were expressed as mean ± standard deviation (SD) and evaluated by Student’s *t*-test. *p*-values less than 0.05 or 0.01 were considered to be statistically significant. Quantitative results were illustrated with GraphPad Prism software (Version 7.00).

## 3. Results and Discussion

### 3.1. Identification of the Microbial Metabolites of Naringin after In Vitro Fecal Fermentation

Metabolites identification was performed in light of the following two points: (I) full understanding of the data obtained with available reference standards, including retention time (RT) and MS/MS fragmentation patterns; (II) reported results associated with the metabolism of naringin and other flavonoids. Herein, a total of 35 metabolites were identified or tentatively characterized in the fermentation samples. Their detailed information, including tentative identifications, molecular formulas, RT, detected mass-to-charge ratios (*m*/*z*) of deprotonated quasi-molecular ion, and corresponding mass errors, as well as the characteristic MS/MS fragment ions, are listed in Table 2.

On the basis of their chemical structures, these metabolites could be categorized into flavonoid metabolites and phenolic catabolites. Compounds in the class of “flavonoid metabolites” contained the characteristic carbon skeleton C6-C3-C6. A total of 29 flavonoid metabolites were characterized after the fecal fermentation. In light of the corresponding reference standards, M1, M14, M16, M18, M19, M27, and M29 were identified as rhoifolin, neoeriocitrin, neohesperidin, naringenin, apigenin, eriodictyol, and hesperetin, respectively. For most other metabolites, due to the unavailability of corresponding commercial standards, they were identified on the basis of data obtained with mentioned reference compounds and the published literature.

The MS/MS fragmentation of detected flavonoid metabolites showed typical retro-Diels–Alder (RDA) reactions, aligned with reported studies [35,36]. Taking deprotonated naringenin as an example, it was fragmented into product ions at *m*/*z* 151 and 119 by RDA reaction, which could be considered as diagnostic product ions for naringenin and its derivates. In particular, the MS/MS fragmentation of apigenin, eriodictyol, hesperetin, and corresponding conjugates also gave the ion signal at *m*/*z* 151, which was yielded by RDA reaction similar to naringenin. Meanwhile, a loss of 120 Da (C_4_H_8_O_4_) was observed in the MS/MS fragmentation of flavonoid-*O*-glucosides, such as naringin, rhoifolin, naringin monoglucoside, and so on. The characteristic loss was proposed as a fragment of the glucose moiety.

M2 and M3 both gave [M-H]^−^ at *m/z* 621, 42 Da (C_2_H_2_O) more than that of naringin, were presumed to be acetylated naringin. Considering that naringin has two binding sites, 4′-OH and 5-OH, for acetyl, these two metabolites could be 4′-*O*-acetyl-naringin or 5-*O*-acetyl-naringin. As shown in Table 2, most of the fragment ion signals of M2 and M3 were coincident, except that M3 has an exclusive fragment ion at *m/z* 408. The product ion at *m/z* 408 could be inferred to be formed by deprotonated 5-*O*-acetyl-naringin losing the B ring and a fragment of the glucose moiety, while 4′-*O*-acetyl-naringin was unlikely to give it. Hence, M2 and M3 were tentatively assigned as 4′-*O*-acetyl-naringin and 5-*O*-acetyl-naringin, respectively. Similarly, M9 and M10 were supposed to be 4′-*O*-methyl-naringin or 5-*O*-methyl-naringin. M9 showed the product ions at *m/z* 459 and *m/z* 151, while M10 did not. The characteristic ion signals of M9 could be explained by the cleavage of bonds 1 and 3 in the C ring of 4′-*O*-methyl-naringin. Accordingly, M10 was proposed to be 5-*O*-methyl-naringin.

M25 and M26 exhibited the same [M-H]^−^ with M27 (eriodictyol, i.e., 3′-hydroxy-naringenin) and were proposed to be hydroxyl naringenin. Through the same RDA reaction occurring on the bonds 1 and 3 in C ring, M25 and M26 gave the product ions at *m/z* 167 and *m/z* 119, while eriodictyol formed the fragment ions at *m/z* 151 and *m/z* 135. Based on the difference in MS/MS fragmentation pattern, M25 and M26 were inferred to be 6/8-hydroxyl-naringenin. Combined with reported relative retention time data [37], M25 and M26 were tentatively characterized as carthamidin (6-hydroxy-naringenin) and isocarthamidin (8-hydroxy-naringenin), respectively. In another similar case, M13 were presumed to be 6/8-hydroxy-naringin, an isomer of neoeriocitrin. Based on similar correlation between chemical structure and MS/MS fragmentation pattern, M11, M12, M15, M17, M23, M24, and M28 were tentatively identified as the flavonoid metabolites described in Table 2.

M5, M6, M7, and M8 had the same [M-H]^−^ at *m/z* 741, 162 Da (C_6_H_10_O_5_) more than that of naringin, and showed same product ion signals at *m/z* 271 and *m/z* 151. Several studies [38,39] have reported the synthesis of naringin glycosides through *Bacilus* species-mediated transglucosylation. Herein, these four compounds were proposed as the glucose conjugates of naringin. Given the chemical structure of naringin, the new glucose moiety could bind to either the hydroxyl of flavonoid skeleton (4′-OH, 5-OH) or the hydroxyl of 7-*O*-neohesperidose moiety. Further methodologies are required to determine the exact binding sites for glucose moiety in these four compounds. Similarly, M20, M21, and M22 were inferred to be glucosyl naringenin. In the structure of naringenin, 4′-OH, 5-OH, and 7-OH are the three possible conjunct sites for the glucose group. On the basis of relative retention time reported in previous studies [40,41], M20, M21, and M22 were tentatively identified as naringenin-5-*O*-glucoside, naringenin-7-*O*-glucoside, and naringenin-4′-*O*-glucoside, respectively.

Phenolic catabolites were the downstream metabolites that flowed from the ring fission of the flavonoid skeleton. As shown in Table 2, a total of six phenolic catabolites were detected in the fermentation samples. Compared with authentic standards, M30, M31, and M33 were unambiguously identified as *p*-coumaric acid, caffeic acid, and 3-(4′-hydroxyphenyl)propionic acid, respectively. M32, M34, and M35 were presumed to be cinnamic acid, 3-(3′, 4′-dihydroxyphenyl)propionic acid, and 3-phenylpropionic acid based on their MS/MS fragmentation patterns and reported results [23,42]. Due to carrying the carboxyl group, these phenolic catabolites all gave characteristic product ions with the neutral loss of CO_2_ (44 Da).

### 3.2. Biotransformation of Naringin during In Vitro Fecal Fermentation

**Figure 1:**
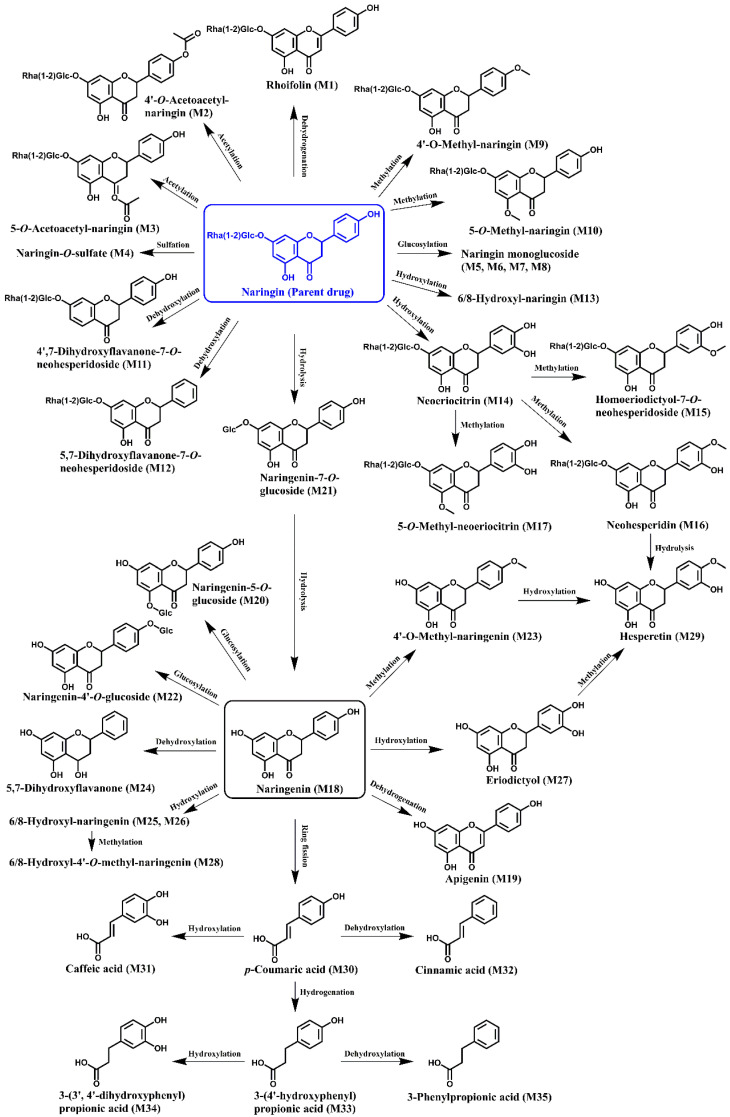
Potential pathways for the metabolism of naringin by rat gut microbiota. The numbers of metabolites in brackets corresponds to those in Table 1.

In this work, microbial-mediated degradation of naringin gave rise to a number of metabolites. On the basis of identified metabolites listed in Table 2, naringin was considered to be engaged in a series of microbial-mediated biotransformations, including dehydrogenation, acetylation, sulfation, glucosylation, methylation, dehydroxylation, hydroxylation, and hydrolysis. With the successive loss of a rhamnose and a glucose moiety, naringin was hydrolyzed to the aglycone naringenin. Generated naringenin was subsequently involved in dehydrogenation, glucosylation, dehydroxylation, hydroxylation, and methylation. Meanwhile, naringenin undergo ring fission and yield *p*-coumaric acid, which was further employed in hydrogenation, hydroxylation, and dehydroxylation, giving rise to several phenolic catabolites. The proposed biotransformation pathways of naringin during in vitro fecal fermentation are illustrated in Figure 1.

To date, human-gut-microbiota-mediated metabolism of naringin has been investigated in several studies. Using the HPLC method, Xiong et al. [26] determined the degradation profile of naringin co-incubated with feces of healthy people, and found that naringin was extensively metabolized into naringenin. With the extension of time, the concentration of naringenin in the incubation system also gradually decreased, suggesting that naringenin could be further degraded by fecal microorganisms, but the corresponding downstream metabolites were not identified. Zou et al. [27] investigated the human intestinal microbial metabolism of naringin with LC-MS/MS technology. Naringin was found to be metabolized into naringenin and further degraded into 3-(4′-hydroxyphenyl)propionic acid. The ability of human intestinal microorganisms to mediate naringenin metabolism to produce 3-(4′-hydroxyphenyl)propionic acid showed significant individual differences. In another study, Chen et al. [28] detected the human-intestinal-microbes-associated metabolic profile of naringin with stable isotope-labeling strategy coupled with high-resolution LC-MS/MS. A total of 13 microbial metabolites were identified, and naringin was found to undergo extensive phase I metabolism mediated by human intestinal microbes. In this study, rat intestinal microbial metabolism of naringin was first investigated. Thanks to more available reference standards and high-resolution UFLC-Q-TOF-MS/MS system, a total of 35 metabolites were detected in the fermentation samples, far more than in previous studies. Based on these metabolites, more comprehensive microbial-mediated naringin metabolic pathways were proposed, as shown in Figure 1.

### 3.3. Quantification of the Microbial Metabolites of Naringin by UHPLC-Q-Trap-MS/MS

Based on the results of metabolite profiling and the availability of corresponding reference standards, naringin and its primary metabolites (including rhoifolin, neoeriocitrin, neohesperidin, naringenin, apigenin, eriodictyol, hesperetin, *p*-coumaric acid, and caffeic acid) were assigned as the target analytes in quantitative analysis. Isoquercitrin was selected as the internal standard (IS). A UHPLC-Q-Trap-MS/MS method was developed and validated for simultaneous determination of these compounds.

Fine peaks were obtained for each target analyte and IS, and no significant interference peaks at their respective RT range were observed. The residual interferences of ten target analytes after the injection of upper limit of quantification sample were all less than 20% of that in LLOQ sample, while that for IS was within 5%. All calibration curves of target analytes achieved acceptable linearity with correction coefficients greater than 0.99 (detailed in Table S1 in the Supplementary Material). The LLOQ of naringin, rhoifolin, neoeriocitrin, neohesperidin, naringenin, apigenin, eriodictyol, hesperetin, *p*-coumaric acid, and caffeic acid were 4.783, 5.185, 5.000, 5.100, 5.350, 0.4715, 0.4464, 0.5510, 0.4804, and 0.5000 ng/mL, respectively. The intra-batch and inter-batch precision (expressed by relative standard deviation, RSD) of LLOQ samples were <12.4% with accuracy (expressed by relative error, RE) in the range of −10.8% to 6.2% for all analytes (Table S2). The intra-batch and inter-batch assay of QC samples (LQC, MQC, HQC) obtained acceptable precision of <11.4%, and accuracy varied from −8.1% to 8.9% for target analytes (Table S2). The IS-normalized matrix factors (IS-MF) of ten target analytes were tested at LQC and HQC concentration levels, and achieved the precision of <5.5% (Table S2). The precision of analytes diluted 20 times was less than 12.4%, with the accuracy varying from −8.4% to 8.7%. In long-term stability, incubation stability, and placement stability tests, the accuracy of LQC and HQC samples were all in the range of −8.3% to 12.7%, and the precision within 13.0% (Table S3). The incubation stability tests showed that naringin was stable when incubated at 37 °C for 12 h, that is to say, naringin would not undergo metabolism in the absence of intestinal microorganisms. All methodology validation assay results were within the acceptable criteria, suggesting that the present method was stable and reliable for simultaneous determination of the abovementioned target analytes in rat fecal fermentation samples.

**Figure 2:**
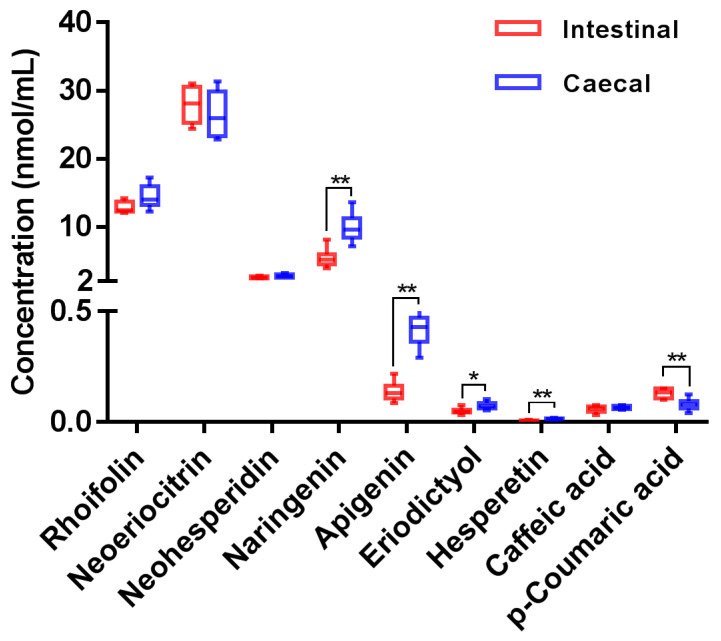
Measured concentrations of target analytes in rat fecal fermentation samples (compared with intestinal contents group; * *p* < 0.05, ** *p* < 0.01).

The validated method was successfully applied to the quantitative determination of naringin and its primary microbial metabolites in rat fecal fermentation samples. Measured concentrations of target analytes are plotted in Figure 2 (detailed in Table S4 in the Supplementary Material). As shown in Figure 2, whether incubated with intestinal or caecal contents, the abundances of rhoifolin, neoeriocitrin, neohesperidin, and naringenin in fermentation samples were much higher than those of other microbial metabolites. It should be noted that the concentrations of some metabolites yielded by incubation with intestinal or caecal contents were significantly different. For example, compared with that in the intestinal contents group, the concentrations of naringenin and apigenin in caecal contents group were significantly higher, while the concentration of *p*-coumaric acid was significantly lower. These results suggest that there was a difference in the capacity of degrading naringin between intestinal or caecal microbiota, probably associated with the community diversity at different locations [43,44]. Li et al. [45] investigated the regional differences of microbiota along the gastrointestinal tract in rat, and found that the community diversity was closely related to biogeographic location. Lactate-producing bacteria (including *Lactobacillus*, *Turicibacter*, and *Streptococcus*) dominated the bulk of small-intestinal microbiota, while a greater proportion of anaerobic Lachnospiraceae and Ruminococcaceae were found to be the core microbiota in the large intestine.

After the fermentation, the total molarity of naringin and its nine microbial metabolites in intestinal contents group and caecal contents group were 334.3 and 363.4 nmol/mL, which were equivalent to 66.9% and 72.7% of the original naringin input. These results suggest that a considerable amount of naringin was metabolized into other metabolites. As shown in Table 2, in addition to the nine compounds quantified, a series of other microbial metabolites were also detected in the fermentation samples. To quantify these metabolites, fermentation samples were further detected in full scan mode and the optimized chromatographic separation conditions of the above UHPLC-Q-Trap-MS/MS method. Acquired peak area data revealed that acetylated, methylated, dehydroxylated, and hydroxylated metabolites of naringin were also abundant in the fermentation samples. Subsequently, the MRM parameters for these abundant metabolites were optimized as follows: *m/z* 621.2→271.1 (DP: −120 V, CE: −45 eV) for acetylated naringin (M2, M3), *m/z* 593.2→285.1 (DP: −80 V, CE: −35 eV) for methylated naringin (M9, M10), *m/z* 563.2→255.1 (DP: −120 V, CE: −45 eV) for dehydroxylated naringin (M11/M12), and *m/z* 595.1→287.1 (DP: −80 V, CE: −35 eV) for hydroxylated naringin (M13). However, due to the unavailability of corresponding authentic standards, these metabolites were quantified using the calibration curves of corresponding structural analogues. Specifically, naringin was used to quantify its acetylated, methylated, and dehydroxylated metabolites, while neoeriocitrin was used to quantify hydroxylated naringin. Measured concentrations of these metabolites are listed in Table S4 in the Supplementary Material. Taking these metabolites into account, the overall recovery of naringin in the intestinal contents group and caecal contents group after incubation reached 86.5% and 92.1%, respectively.

To date, two studies [27,46] have quantified a microbial metabolite of naringin in human fecal fermentation samples, i.e., 3-(4′-hydroxyphenyl)propionic acid, which is also detected in this study. Herein, a rapid UHPLC-Q-Trap-MS/MS method was firstly established for simultaneous determination of nine microbial metabolites derived from naringin. Moreover, six other metabolites with high abundance in fermentation samples were also quantified with constructed calibration curves of authentic standards with similar structures. This work provides a feasible quantitative method for other studies concerning the microbial metabolism of flavonoids.

In this study, the metabolic profiles of naringin mediated by rat gut microbiota were investigated qualitatively and quantitatively; however, information concerning the association between microbial species and specific metabolic pathways is still limited. Characterization of the compositional profile of microbiota is essential to confirm mentioned association. The integration of in vitro fermentation and sequencing methods (e.g., 16S rDNA) should be a feasible strategy to clarify the correlation between certain microbial species and specific metabolic pathways.

### 3.4. Effect of Fecal Fermentation on the Antioxidant Capacity

**Figure 3:**
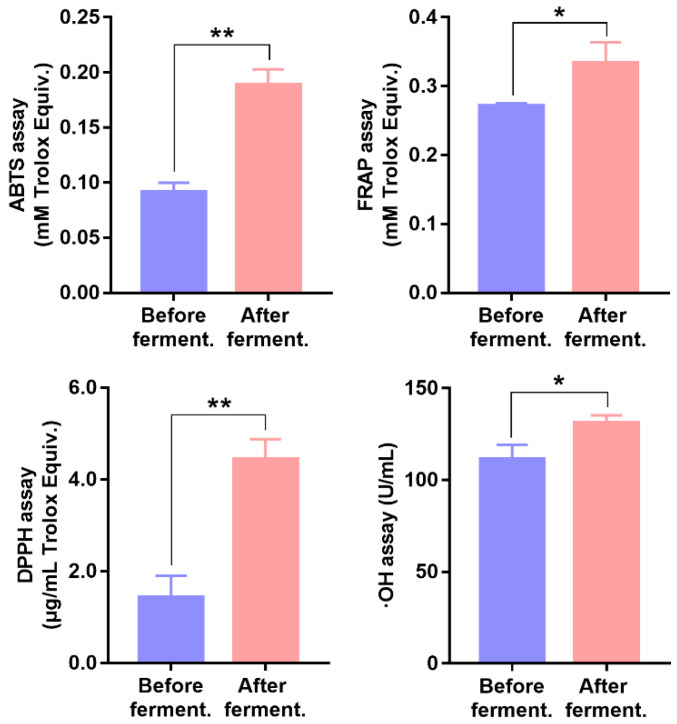
Antioxidant capacity of pre-fermented and post-fermented working solutions of cecal contents group (compared with intestinal pre-fermented working solution; * *p* < 0.05, ** *p* < 0.01).

In this work, ABTS, FRAP, DPPH, and ∙OH assays were applied to evaluate the TAOC changes before and after fecal fermentation. Obtained results are plotted in Figure 3. Compared with the pre-fermented working solution, the post-fermented working solution showed a significant increase in the TAOC values. In other words, fermentation-associated microbial metabolites exhibited higher antioxidant activity than original naringin. On the one hand, naringin was metabolized into some flavonoid compounds with polyhydroxyl groups and higher antioxidant activity [9]. On the other hand, gut-microbiota-related gastrointestinal digestion led to better overall bioavailability and bioaccessibility of naringin [14,17]. A number of studies [47,48,49] have indicated that gastrointestinal tract is constantly being challenged by free radicals, including diet-derived oxidants and endogenous reactive oxygen species. Therefore, the presence of fermentation-associated metabolites could help to relieve oxidative stress in the gastrointestinal tract.

## 4. Conclusions

In summary, rat-fecal-microbiota-mediated metabolism of naringin and its effect on the antioxidant capacity was investigated in this work. UFLC-Q-TOF-MS/MS-based metabolite identification results showed that rat fecal microbiota could metabolize naringin into a number of metabolites, including 29 flavonoid metabolites and 6 phenolic catabolites. Naringin was found to undergo a series of microbial-mediated biotransformations, and corresponding metabolic pathways were proposed. Moreover, a UHPLC-Q-Trap-MS/MS method was developed for simultaneous determination of naringin and its metabolites in fecal fermentation samples. Rhoifolin, neoeriocitrin, neohesperidin, and naringenin, as well as the methylated and hydroxylated metabolites of naringin, were detected as the primary microbial metabolites. Further antioxidant capacity assays indicated that fermentation-associated microbial metabolites showed higher antioxidant activity than original naringin. The present results provide new insights in understanding the microbial metabolism and in vivo antioxidant capacity of naringin.

## Figures and Tables

**Table 1:** The optimized MS/MS transitions, declustering potential (DP), and collision energy (CE) parameters for target analytes.

No.	Analytes	Q1 Mass (Da)	Q3 Mass (Da)	DP (eV)	CE (eV)
1	Naringin	579.1	271.1	−45	−120
2	Rhoifolin	577.1	269.0	−49	−46
3	Neoeriocitrin	595.1	459.1	−36	−98
4	Neohesperidin	609.1	301.1	−39	−131
5	Naringenin	270.9	150.9	−24	−44
6	Apigenin	269.0	151.0	−33	−48
7	Eriodictyol	286.9	135.0	−38	−56
8	Hesperetin	301.0	163.9	−32	−134
9	*p*-Coumaric acid	163.0	119.1	−15	−83
10	Caffeic acid	179.0	135.0	−35	−80
11	Isoquercitrin (IS)	463.1	299.9	−36	−32

**Table 2:** UHPLC-Q-TOF-MS/MS-based identification of the microbial metabolites of naringin after in vitro fecal fermentation.

No.	Identified Compounds	Formula	RT (min)	[M-H]^−^ (Error, ppm)	Fragment Ions *^a^*
Parent drug	Naringin *^b^*	C_27_H_32_O_14_	11.5	579.1723 (0.6)	459.1120[M-H-C_8_H_8_O]^−^, 339.0710[M-H-C_8_H_8_O-C_4_H_8_O_4_]^−^, 313.0708[M-H-C_8_H_8_O-Rha]^−^, 271.0590[M-H-Rha-Glc]^−^, 177.0186[M-H-Rha-Glc-C_6_H_6_O]^−^, 151.0023[M-H-Rha-Glc-C_8_H_8_O]^−^, 119.0493[M-H-Rha-Glc-C_7_H_4_O_4_]^−^
M1	Rhoifolin *^b^*	C_27_H_30_O_14_	12.1	577.1571 (1.4)	431.0987[M-H-Rha]^−^, 413.0871[M-H-Rha-H_2_O]^−^, 311.0550[M-H-Rha-C_4_H_8_O_4_]^−^, 269.0447[M-H-Rha-Glc]^−^
M2	4′-*O*-Acetyl-naringin	C_29_H_34_O_15_	12.4	621.1808 (−2.7)	579.1741[M-H-C_2_H_2_O]^−^, 501.1221[M-H-C_4_H_8_O_4_]^−^, 459.1327[M-H-C_4_H_8_O_4_-C_2_H_2_O]^−^, 339.0740[M-H-C_4_H_8_O_4_-C_2_H_2_O-C_8_H_8_O]^−^, 271.0594[M-H-C_2_H_2_O-Rha-Glc]^−^, 151.0041[M-H-C_2_H_2_O-Rha-Glc-C_8_H_8_O]^−^
M3	5-*O*-Acetyl-naringin	C_29_H_34_O_15_	13.4	621.1816 (−1.5)	579.1533[M-H-C_2_H_2_O]^−^, 577.3041, 501.1253[M-H-C_4_H_8_O_4_]^−^, 459.1389[M-H-C_4_H_8_O_4_-C_2_H_2_O]^−^, 408.2170[M-H-C_4_H_8_O_4_-C_6_H_5_O]^−^, 339.0658[M-H-C_4_H_8_O_4_-C_2_H_2_O-C_8_H_8_O]^−^, 271.0593[M-H-C_2_H_2_O-Rha-Glc]^−^, 151.0030[M-H-C_2_H_2_O-Rha-Glc-C_8_H_8_O]^−^, 119.0434[M-H-C_2_H_2_O-Rha-Glc-C_7_H_4_O_4_]^−^
M4	Naringin-*O*-sulfate	C_27_H_32_O_17_S	10.8	659.1339 (4.8)	596.1722, 579.1683[M-H-SO_3_]^−^, 459.1150[M-H-SO_3_-C_8_H_8_O]^−^, 387.0670, 351.0199[M-H-Rha-Glc]^−^, 271.0629[M-H-SO_3_-Rha-Glc]^−^, 151.0046[M-H-SO_3_-Rha-Glc-C_8_H_8_O]^−^
M5	Naringin monoglucoside	C_33_H_42_O_19_	9.7	741.2266 (2.4)	579.1736[M-H-Glc]^−^, 459.1147[M-H-Glc-C_8_H_8_O]^−^, 433.1197[M-H-Glc-Rha]^−^, 339.0582[M-H-Glc-C_8_H_8_O-C_4_H_8_O_4_]^−^, 313.0691[M-H-Glc-Rha-C_8_H_8_O]^−^, 271.0646[M-H-Glc-Rha-Glc]^−^, 151.0080[M-H-Glc-Rha-Glc-C_8_H_8_O]^−^
M6	Naringin monoglucoside	C_33_H_42_O_19_	10.1	741.2266 (2.4)	723.3698[M-H-H_2_O]^−^, 621.1735[M-H-C_4_H_8_O_4_]^−^, 459.1375[M-H-C_4_H_8_O_4_-Glc]^−^, 339.0673[M-H-C_4_H_8_O_4_-Glc-C_8_H_8_O]^−^, 271.0598[M-H-Glc-Rha-Glc]^−^, 151.0053[M-H-Glc-Rha-Glc-C_8_H_8_O]^−^
M7	Naringin monoglucoside	C_33_H_42_O_19_	10.3	741.2250 (0.4)	621.1645[M-H-C_4_H_8_O_4_]^−^, 579.1766[M-H-Glc]^−^, 459.1364[M-H-C_4_H_8_O_4_-Glc]^−^, 339.0701[M-H-C_4_H_8_O_4_-Glc-C_8_H_8_O]^−^, 271.0609[M-H-Glc-Rha-Glc]^−^, 151.0057[M-H-Glc-Rha-Glc-C_8_H_8_O]^−^
M8	Naringin monoglucoside	C_33_H_42_O_19_	11.3	741.2253 (0.8)	621.1674[M-H-C_4_H_8_O_4_]^−^, 595.1883[M-H-Rha]^−^, 579.1776[M-H-Glc]^−^, 271.0615[M-H-Glc-Rha-Glc]^−^, 227.0710, 151.0056[M-H-Glc-Rha-Glc-C_8_H_8_O]^−^
M9	4′-*O*-Methyl-naringin	C_28_H_34_O_14_	13.3	593.1866 (−1.6)	473.1440[M-H-C_4_H_8_O_4_]^−^, 459.1134[M-H-C_9_H_10_O]^−^, 447.1294[M-H-Rha]^−^, 431.1334[M-H-C_9_H_10_O-CO]^−^, 387.1080[M-H-Rha-C_2_H_4_O_2_]^−^, 327.0868[M-H-C_4_H_8_O_4_-Rha]^−^, 285.0756[M-H-Rha-Glc]^−^, 270.0534[M-H-Rha-Glc-CH_3_]^−^, 241.0868, 164.0120, 151.0039[M-H-Rha-Glc-C_9_H_10_O]^−^
M10	5-*O*-Methyl-naringin	C_28_H_34_O_14_	14.3	593.1857 (−3.2)	513.2740, 473.1496[M-H-C_4_H_8_O_4_]^−^, 327.0802[M-H-C_4_H_8_O_4_-Rha]^−^, 285.0746[M-H-Rha-Glc]^−^, 241.0898
M11	4′,7-Dihydroxyflavanone-7-*O*-neohesperidoside	C_27_H_32_O_13_	13.3	563.1774 (0.7)	443.1395[M-H-C_8_H_8_O]^−^, 297.0875[M-H-C_8_H_8_O-Rha]^−^, 255.0680[M-H-Rha-Glc]^−^, 211.0774
M12	5,7-Dihydroxyflavanone-7-*O*-neohesperidoside	C_27_H_32_O_13_	13.5	563.1780 (1.8)	459.1256[M-H-C_8_H_8_]^−^, 443.1233[M-H-C_4_H_8_O_4_]^−^, 339.0747[M-H-C_8_H_8_-C_4_H_8_O_4_]^−^, 271.0623, 151.0023[M-H-C_8_H_8_-Rha-Glc]^−^, 119.0526
M13	6/8-Hydroxyl-naringin	C_27_H_32_O_15_	10.3	595.1671 (0.4)	475.1084[M-H-C_8_H_8_O]^−^, 431.0978[M-H-C_8_H_8_O-CO_2_]^−^, 287.0580[M-H-Rha-Glc]^−^, 269.0453, 166.9985[M-H-Rha-Glc-C_8_H_8_O]^−^, 153.0195, 139.0039[M-H-Rha-Glc-C_8_H_8_O-CO]^−^, 119.04895[M-H-Rha-Glc-C_7_H_4_O_5_]^−^
M14	Neoeriocitrin *^b^*	C_27_H_32_O_15_	10.7	595.1671 (0.5)	475.1102[M-H-C_4_H_8_O_4_]^−^, 459.1135[M-H-C_8_H_8_O_2_]^−^, 339.0718[M-H-C_8_H_8_O_2_-C_4_H_8_O_4_]^−^, 287.0572[M-H-Rha-Glc]^−^, 235.0254, 193.0152, 151.0039[M-H-Rha-Glc-C_8_H_8_O_2_]^−^, 135.0448[M-H-Rha-Glc-C_7_H_4_O_4_]^−^
M15	Homoeriodictyol-7-*O*-neohesperidoside	C_28_H_34_O_15_	11.6	609.1794 (−4.5)	563.1417[M-H-CO-H_2_O]^−^, 489.1238[M-H-C_4_H_8_O_4_]^−^, 459.1091[M-H-C_9_H_10_O_2_]^−^, 343.0874[M-H -C_4_H_8_O_4_-Rha]^−^, 301.0742[M-H-Rha-Glc]^−^, 235.0208, 151.0023[M-H-Rha-Glc-C_9_H_10_O_2_]^−^, 125.0272
M16	Neohesperidin *^b^*	C_28_H_34_O_15_	11.8	609.1830 (0.8)	489.1373[M-H-C_4_H_8_O_4_]^−^, 403.1013, 343.0823[M-H -C_4_H_8_O_4_-Rha]^−^, 325.0736[M-H-Rha-C_4_H_8_O_4_-H_2_O]^−^, 301.0718[M-H-Rha-Glc]^−^, 286.0494[M-H-Rha-Glc-CH_3_]^−^, 257.0820[M-H-Rha-Glc-CH_3_-HCO]^−^, 242.0581[M-H-Rha-Glc-OCH_2_-HCO]^−^, 164.0127[M-H-Rha-Glc-C_8_H_9_O_2_]^−^, 151.0051[M-H-Rha-Glc-C_9_H_10_O_2_]^−^, 125.0260
M17	5-*O*-Methyl-neoeriocitrin	C_28_H_34_O_15_	12.4	609.1824 (−0.2)	591.2831[M-H-H_2_O]^−^, 445.1171[M-H-H_2_O-Rha]^−^, 364.1490, 301.0718[M-H-Rha-Glc]^−^, 273.0760[M-H-Rha-Glc-CO]^−^, 226.1194, 165.9897[M-H-Rha-Glc-C_8_H_7_O_2_]^−^
M18	Naringenin *^b^*	C_15_H_12_O_5_	13.7	271.0615 (1.0)	227.0697, 177.0190[M-H-C_6_H_6_O]^−^, 151.0028[M-H-C_8_H_8_O]^−^, 119.0495[M-H-C_7_H_4_O_4_]^−^, 107.0134[M-H-C_8_H_8_O-CO_2_]^−^, 93.0339[M-H-C_9_H_6_O_4_]^−^, 83.0127
M19	Apigenin *^b^*	C_15_H_10_O_5_	15.0	269.0454 (−0.6)	225.0552, 201.0555, 151.0031[M-H-C_8_H_6_O]^−^, 117.0342[M-H-C_7_H_4_O_4_]^−^, 107.0132[M-H-C_8_H_6_O-CO_2_]^−^
M20	Naringenin-5-*O*-glucoside	C_21_H_22_O_10_	11.8	433.1145 (1.2)	415.1077[M-H-H_2_O]^−^, 373.0961[M-H-C_2_H_4_O_2_]^−^, 343.0830[M-H-C_3_H_6_O_3_]^−^, 313.0707[M-H-C_4_H_8_O_4_]^−^, 271.0613[M-H-Glc]^−^, 223.0271[M-H-C_3_H_6_O_3_-C_8_H_8_O]^−^, 205.0153[M-H-C_3_H_6_O_3_-C_8_H_8_O-H_2_O]^−^, 151.0029[M-H-Glc-C_8_H_8_O]^−^, 119.0494[M-H-Glc-C_7_H_4_O_4_]^−^, 93.0357[M-H-Glc-C_9_H_6_O_4_]^−^
M21	Naringenin-7-*O*-glucoside	C_21_H_22_O_10_	12.2	433.1140 (−0.1)	415.1071[M-H-H_2_O]^−^, 397.0795[M-H-2H_2_O]^−^, 373.0938[M-H-C_2_H_4_O_2_]^−^, 343.0812[M-H-C_3_H_6_O_3_]^−^, 313.0700[M-H-C_4_H_8_O_4_]^−^, 271.0618[M-H-Glc]^−^, 223.0243[M-H-C_3_H_6_O_3_-C_8_H_8_O]^−^, 151.0026[M-H-Glc-C_8_H_8_O]^−^, 119.0492[M-H-Glc-C_7_H_4_O_4_]^−^, 107.0116[M-H-Glc-C_8_H_8_O-CO_2_]^−^
M22	Naringenin-4′-*O*-glucoside	C_21_H_22_O_10_	12.9	433.1140 (0.1)	313.0765[M-H-C_4_H_8_O_4_]^−^, 271.0606[M-H-Glc]^−^, 151.0050[M-H-Glc-C_8_H_8_O]^−^, 119.0511[M-H-Glc-C_7_H_4_O_4_]^−^, 93.0322[M-H-Glc-C_9_H_6_O_4_]^−^
M23	4′-*O*-Methyl-naringenin	C_16_H_14_O_5_	16.5	285.0771 (0.9)	270.0542[M-H-CH_3_]^−^, 243.0665[M-H-C_2_H_2_O]^−^, 227.0348, 215.0727, 201.0563, 175.0774, 164.0121, 151.0034[M-H-C_9_H_10_O]^−^, 136.0159, 108.0214
M24	5,7-Dihydroxyflavanone	C_15_H_12_O_4_	16.7	255.0665 (0.9)	213.0523[M-H-C_2_H_2_O]^−^, 171.0408, 151.0029[M-H-C_8_H_8_]^−^, 145.0664, 107.0127[M-H-C_8_H_8_-CO_2_]^−^, 83.0114
M25	6-Hydroxyl-naringenin	C_15_H_12_O_6_	11.9	287.0565 (1.4)	259.0613[M-H-CO]^−^, 243.0673, 181.0136[M-H-C_7_H_6_O]^−^, 166.9989[M-H-C_8_H_8_O]^−^, 153.0189[M-H-C_7_H_6_O-CO]^−^, 139.0025[M-H-C_8_H_8_O-CO]^−^, 119.0494[M-H-C_7_H_4_O_5_]^−^, 111.0085, 95.0139
M26	8-Hydroxyl-naringenin	C_15_H_12_O_6_	12.2	287.0564 (1.0)	259.0616[M-H-CO]^−^, 193.0131[M-H-C_6_H_6_O]^−^, 181.0152[M-H-C_7_H_6_O]^−^, 166.9982[M-H-C_8_H_8_O]^−^, 153.0195[M-H-C_7_H_6_O-CO]^−^, 139.0032[M-H-C_8_H_8_O-CO]^−^, 123.0055[M-H-C_8_H_8_O-CO_2_]^−^, 119.0497[M-H-C_7_H_4_O_5_]^−^, 111.0071, 95.0135
M27	Eriodictyol *^b^*	C_15_H_12_O_6_	12.8	287.0565 (1.3)	151.0027[M-H-C_8_H_8_O_2_]^−^, 135.0445[M-H-C_7_H_4_O_4_]^−^, 125.0231, 107.0136[M-H-C_8_H_8_O_2_-CO_2_]^−^, 83.0138
M28	6/8-Hydroxyl-4′-*O*-methyl-naringenin	C_16_H_14_O_6_	11.2	301.0713 (−1.5)	257.0785[M-H-CH_3_-HCO]^−^, 213.0930, 195.0816[M-H-C_7_H_6_O]^−^, 107.0501[M-H-C_9_H_6_O_5_]^−^, 93.0351[M-H-C_9_H_6_O_5_-CH_2_]^−^
M29	Hesperetin *^b^*	C_16_H_14_O_6_	14.0	301.0712 (−1.9)	286.0525[M-H-CH_3_]^−^, 257.0766[M-H-CH_3_-HCO]^−^, 242.0533[M-H-OCH_2_-HCO]^−^, 224.0465, 199.0381, 164.0103[M-H-C_8_H_9_O_2_]^−^, 151.0021[M-H-C_9_H_10_O_2_]^−^, 135.0405, 108.0206
M30	*p*-Coumaric acid *^b^*	C_9_H_8_O_3_	10.9	163.0407 (3.8)	119.0495[M-H-CO_2_]^−^, 93.0344[M-H-CO_2_-C_2_H_2_]^−^
M31	Caffeic acid *^b^*	C_9_H_8_O_4_	9.5	179.0351 (0.9)	135.0448[M-H-CO_2_]^−^, 107.0493, 89.0399
M32	Cinnamic acid	C_9_H_8_O_2_	11.2	147.0451 (−0.1)	103.0562[M-H-CO_2_]^−^
M33	3-(4′-Hydroxyphenyl)propionic acid *^b^*	C_9_H_10_O_3_	10.9	165.0558 (0.6)	121.0657[M-H-CO_2_]^−^, 119.0491[M-H-HCOOH]^−^, 106.0412[M-H-CO_2_-CH_3_]^−^, 96.9628
M34	3-(3′, 4′-Dihydroxyphenyl)propionic acid	C_9_H_10_O_4_	7.9	181.0506 (−0.1)	163.0400[M-H-H_2_O]^−^, 135.0475[M-H-H_2_O-CO]^−^, 119.0469[M-H-H_2_O-CO_2_]^−^, 107.0490[M-H-H_2_O-2CO]^−^
M35	3-Phenylpropionic acid	C_9_H_10_O_2_	13.6	149.0609 (0.4)	131.0104[M-H-H_2_O]^−^, 105.0679[M-H-CO_2_]^−^

*^a^* The losses are as follows: Rha = rhamnose moiety; Glc = glucose moiety. *^b^* Confirmation in comparison with authentic standards.

## Data Availability

Data is contained within the article.

## Supplementary Materials

The following supporting information can be downloaded at: https://www.mdpi.com/article/10.3390/nu14183765/s1, Table S1: The calibration curves, correlation coefficients (r), and linear ranges of ten analytes (naringin, rhoifolin, neoeriocitrin, neohesperidin, naringenin, apigenin, eriodictyol, hesperetin, *p*-coumaric acid, and caffeic acid); Table S2: Intra-batch and inter-batch precision, accuracy, and IS-normalized matrix factor (IS-MF) of target analytes in rat fecal fermentation samples; Table S3: Stability of target analytes in rat fecal fermentation samples under different conditions (*n* = 3); Table S4: Measured concentrations (nmol/mL) of target analytes in rat fecal fermentation samples.
