# Supplementary material for: Microbial Metabolism of Naringin and the Impact on Antioxidant Capacity
Source: Nutrients. 2022 Sep 13;14(18):3765. doi: 10.3390/nu14183765 (PMC9502552; doi:10.3390/nu14183765)
Supplement: Supplementary file 1 [file nutrients-14-03765-s001.zip › nutrients-1889899-supplementary.pdf]

Table S1. The calibration curves, correlation coefficients (r), and linear ranges of ten analytes.

No.	Analytes	Calibration curves	r	Linear ranges (ng/mL)
1	Naringin	$y=6.48438\times10^{-5}x+0.02807$	0.99256	4.783~956.7
2	Rhoifolin	$y=2.01783\times10^{-4}x+0.00293$	0.99755	5.185~1037
3	Neoeriocitrin	$y=7.12302\times10^{-6}x+2.07757\times10^{-5}$	0.99723	5.000~1000
4	Neohesperidin	$y=1.08542\times10^{-4}x+2.69833\times10^{-4}$	0.99836	5.100~1020
5	Naringenin	$y=4.14973\times10^{-4}x+0.01161$	0.99890	5.350~1070
6	Apigenin	$y=2.09550\times10^{-4}x+1.18562\times10^{-4}$	0.99845	0.4715~94.30
7	Eriodictyol	$y=4.46139\times10^{-4}x+1.64672\times10^{-4}$	0.99942	0.4464~89.28
8	Hesperetin	$y=1.46065\times10^{-4}x+1.72990\times10^{-5}$	0.99877	0.5510~110.2
9	*p*-Coumaric acid	$y=1.09446\times10^{-4}x+0.00128$	0.99395	0.4804~96.09
10	Caffeic acid	$y=6.29794\times10^{-5}x+1.12458\times10^{-5}$	0.99796	0.5000~100.0

Table S2. Intra-batch and inter-batch precision, accuracy and IS-normalized matrix factor (IS-MF) of target analytes in rat fecal fermentation samples.

Analytes	Conc. (ng/mL)	Intra-batch (n=6)		Inter-batch (n=6*3)		RSD% of IS-MF (n=3*6)
		RSD%	RE%	RSD%	RE%	
Naringin	4.783	8.9	4.5	10.0	2.4	—
	14.35	8.1	-0.9	7.1	2.1	0.5
	143.5	7.2	1.8	9.7	-1.6	—
	717.5	6.2	1.0	7.2	-0.3	3.2
Rhoifolin	5.185	7.5	-0.4	8.2	-2.4	—
	15.56	4.8	-6.2	8.5	-2.4	3.7
	155.6	11.1	1.1	9.5	0.5	—
	777.8	4.3	3.4	6.9	1.7	4.8
Neoeriocitrin	5.000	10.8	3.2	11.4	1.1	—
	15.00	6.5	-0.5	8.2	-1.7	3.0
	150.0	7.1	1.9	7.4	2.2	—
	750.0	3.2	-3.2	7.1	-3.4	5.1
Neohesperidin	5.100	2.5	-6.5	7.4	-3.6	—
	15.30	5.1	1.1	8.3	-1.9	5.1
	153.0	9.8	3.9	7.9	0.9	—
	765.0	4.8	0.1	6.3	-0.5	4.3
Naringenin	5.350	9.8	-0.4	9.5	1.3	—
	16.05	10.8	2.8	9.1	-0.9	1.1
	160.5	6.9	6.2	7.3	3.0	—
	802.5	4.7	-0.3	5.4	-3.6	3.3
Apigenin	0.4715	9.2	0.6	10.2	2.6	—
	1.415	4.9	-8.1	5.8	-5.9	2.1
	14.15	9.0	-0.9	8.1	-0.7	—
	70.73	3.8	-2.0	3.5	-2.3	3.9
Eriodictyol	0.4464	8.3	-4.7	7.1	-6.6	—
	1.339	11.4	1.4	9.6	2.3	2.4

	13.39	4.8	8.9	7.0	4.6	—
	66.96	9.3	-0.3	6.3	0.2	3.7
Hesperetin	0.5510	5.6	-10.8	5.2	-9.2	—
	1.653	7.2	-3.0	5.7	-1.6	5.0
	16.53	7.0	3.5	6.5	3.9	—
	82.65	3.5	1.0	3.3	1.8	2.8
p-Coumaric acid	0.4804	12.4	4.3	11.2	0.1	—
	1.441	9.4	3.2	10.3	0.7	0.5
	14.41	9.4	-2.0	7.8	-1.1	—
	72.07	5.2	-0.6	5.6	-3.1	5.5
Caffeic acid	0.5000	7.7	6.2	9.0	4.0	—
	1.500	6.1	1.6	7.5	1.3	5.3
	15.00	8.1	-1.9	6.2	-1.8	—
	75.00	5.2	2.4	4.7	1.2	3.7

Table S3. Stability of target analytes in rat fecal fermentation samples under different conditions (n=3).

Analytes	Conc. (ng/mL)	Long term (-70 ℃, 1 month)		Incubation (37 ℃, 12 h)		Placement in autosampler (15 ℃, 24 h)		Dilution integrity (20 times)	
		RSD%	RE%	RSD%	RE%	RSD%	RE%	RSD%	RE%
Naringin	14.35	10.3	-2.2	10.8	-5.6	10.0	3.2	—	—
	717.5	7.3	3.8	4.4	-2.5	4.5	6.3	8.4	-3.9
Rhoifolin	15.56	5.7	1.5	8.0	-5.1	5.7	-7.8	—	—
	777.8	10.7	-3.7	7.3	7.9	5.6	3.5	11.0	-0.7
Neoeriocitrin	15.00	11.4	2.5	7.0	4.7	13.0	4.5	—	—
	750.0	5.5	1.5	9.5	-0.2	3.7	3.8	6.2	-8.4
Neohesperidin	15.30	6.8	-7.8	5.5	5.4	6.4	3.6	—	—
	765.0	3.3	4.6	9.8	0.3	3.4	10.4	12.4	-3.3
Naringenin	16.05	2.6	-1.1	6.1	-3.8	8.6	-6.3	—	—
	802.5	0.4	1.8	5.2	-4.5	4.2	4.7	9.6	-3.3
Apigenin	1.415	3.0	-6.7	7.5	-3.9	5.5	-1.7	—	—
	70.73	3.1	3.6	5.2	-3.4	0.3	5.3	9.0	-5.2
Eriodictyol	1.339	6.8	-3.4	2.1	-0.2	9.5	5.7	—	—
	66.96	3.8	8.6	1.8	2.5	4.6	-1.9	6.1	8.7
Hesperetin	1.653	3.2	-6.8	8.5	-6.4	8.3	6.3	—	—
	82.65	1.5	6.0	3.3	-1.2	0.7	4.6	9.1	-2.1
p-Coumaric acid	1.441	8.1	-2.2	2.1	12.7	7.6	5.1	—	—
	72.07	4.4	-1.3	4.4	-8.3	1.6	4.7	1.7	-5.6
Caffeic acid	1.500	4.3	9.0	1.9	1.3	7.7	-6.6	—	—
	75.00	7.6	1.6	1.6	-0.8	3.2	-1.4	10.2	-0.3

Table S4. Measured concentrations (nmol/mL) of target analytes in rat fecal fermentation samples.

Concentration (nmol/mL)	Intestinal contents group							Caecal contents group						
	1	2	3	4	5	6		1	2	3	4	5	6	
Naringin	265.1	295.6	259.7	276.5	346.4	267.7	285.2	303.1	278.6	297.8	320.9	330.2	324.3	309.2
Rhoifolin	12.24	12.50	13.49	12.03	14.20	12.29	12.79	13.54	14.08	12.23	15.51	13.84	17.30	14.42
Neoeriocitrin	31.06	24.41	30.40	25.52	29.17	27.15	27.95	22.88	25.85	23.46	26.11	31.39	29.31	26.50
Neohesperidin	2.803	2.462	2.561	2.552	2.679	2.397	2.576	2.637	2.588	2.557	2.812	2.940	3.194	2.788
Naringenin	5.265	3.925	4.711	5.092	8.128	5.173	5.382	8.861	8.808	13.58	10.49	7.096	10.23	9.844**
Apigenin	0.1334	0.0863	0.1483	0.1304	0.2181	0.1152	0.1386	0.4245	0.3865	0.5623	0.4393	0.2914	0.4339	0.4230**
Eriodictyol	0.0483	0.0328	0.0457	0.0464	0.0756	0.0469	0.0493	0.0680	0.0647	0.1061	0.0806	0.0539	0.0732	0.0744*
Hesperetin	0.0052	0.0036	0.0051	0.0056	0.0092	0.0054	0.0057	0.0142	0.0126	0.0207	0.0168	0.0102	0.0154	0.0150**
p-Coumaric acid	0.1539	0.1524	0.1201	0.1092	0.1029	0.1510	0.1316	0.0674	0.0844	0.0403	0.0772	0.0801	0.1269	0.0794**
Caffeic acid	0.0523	0.0675	0.0325	0.0536	0.0764	0.0670	0.0582	0.0547	0.0661	0.0729	0.0608	0.0609	0.0757	0.0652
4’-*O*-Acetyl-naringin (M2)	1.181	0.9659	1.017	0.9967	1.132	0.9952	1.048	0.1156	0.1744	0.0909	0.1602	0.2218	0.1537	0.1530**
5-*O*-Acetyl-naringin (M3)	0.4368	0.2827	0.4266	0.3139	0.4302	0.3817	0.3787	0.0000	0.0083	0.0000	0.0123	0.1104	0.0232	0.0257**
4’-*O*-Methyl-naringin (M9)	1.197	1.117	1.389	1.182	1.377	1.168	1.238	1.238	1.269	1.272	1.232	1.127	1.324	1.244
5-*O*-Methyl-naringin (M10)	37.46	37.89	44.00	36.68	36.94	36.06	38.17	39.98	38.62	37.66	44.03	42.90	44.81	41.33
Dehydroxylated naringin (M11/M12)	1.249	1.270	1.393	1.267	1.554	1.235	1.328	1.291	1.337	1.199	1.480	1.356	1.448	1.352
6/8-Hydroxyl-naringin (M13)	58.35	49.33	64.94	47.61	63.81	52.74	56.13	49.66	48.62	48.48	54.96	56.28	59.62	52.94
Total	416.7	430.1	424.4	410.1	506.3	407.7	432.5	443.9	420.6	439.1	478.4	488.0	492.4	460.4
Mean	432.5							460.4						

Note: Compared with intestinal contents group, * P<0.05, ** P<0.01.
